# Low-Cost Prototype to Automate the 3D Digitization of Pieces: An Application Example and Comparison

**DOI:** 10.3390/s21082580

**Published:** 2021-04-07

**Authors:** Ramón González-Merino, Elena Sánchez-López, Pablo E. Romero, Jesús Rodero, Rafael E. Hidalgo-Fernández

**Affiliations:** 1 Technology Centre of Metal-Mechanical and Transport, Department of Visual Computing, 23700 Linares, Spain; r.gonzalez@cetemet.es (R.G.-M.); j.rodero@cetemet.es (J.R.); 2 Department of Graphic and Geomatic Engineering, Campus of Rabanales, University of Córdoba, 14014 Córdoba, Spain; ig1hifer@uco.es; 3 Department of Mechanical Engineering, Campus of Rabanales, University of Córdoba, 14014 Córdoba, Spain; p62rocap@uco.es

**Keywords:** 3D scanner, photogrammetry, design, 3D printing, additive manufacturing

## Abstract

This work is aimed at describing the design of a mechanical and programmable 3D capturing system to be used by either 3D scanner or DSLR camera through photogrammetry. Both methods are widely used in diverse areas, from engineering, architecture or archaeology, up to the field of medicine; but they also entail certain disadvantages, such as the high costs of certain equipment, such as scanners with some precision, and the need to resort to specialized operatives, among others. The purpose of this design is to create a robust, precise and cost-effective system that improves the limitations of the present equipment on the market, such as robotic arms or rotary tables. For this reason, a preliminary study has been conducted to analyse the needs of improvement, later, we have focused on the 3D design and prototyping. For its construction, there have been used the FDM additive technology and structural components that are easy to find in the market. With regards to electronic components, basic electronics and Arduino-based 3D printers firmware have been selected. For system testing, the capture equipment consists of a Spider Artec 3D Scanner and a Nikon 5100 SLR Camera. Finally, 3D models have been developed by comparing the 3D meshes obtained by the two methods, obtaining satisfactory results.

## 1. Introduction

Nowadays, 3D scanning and photogrammetry techniques are very useful to build three-dimensional digital models in different fields such as engineering, where they are applied in the remodelling and maintenance of industrial plants [1] or band vehicle modelling [2]. In architecture, for the study of structures [3], creating models of buildings [4], or seismic studies [5]; in archaeology, to compare current and ancient landscapes [6], in reconstruction processes [7], and maritime archaeology [8]; or in medicine, diagnosis of pathologies [9], anatomical modelling [10,11], virtual reality [12] or odontology [13] among others applications.

With regards to the principle of operation of these techniques, it must be noted that it is different; 3D scanner creates point cloud from the surface of the object and photogrammetry determines the geometric and spatial properties of objects from photographic images, i.e., 2D images are used to create a 3D model.

Both 3D scanning and photogrammetry have a wide working range, depending on the dimensions of the model to be scanned. In this article we are going to focus on the close range in both photogrammetry and 3D scanning [14]. The term close range refers to a range that covers approximately from 0.1 to 200 m, which we will reduce in our system to pieces of 100 to 400 mm, expecting accuracies of 0.5 mm. Our prototype is primarily intended for archaeological, medical and engineering applications, and with slight modifications, accuracies for industrial use can be achieved [15,16,17].

The laser triangulation scanner (close range laser scanning or CRLS) [18] can document a close range of shapes in three dimensions with great detail and precision. A 3D scanner can measure the distance of many points, obtaining angular data, being able to calculate the three-dimensional coordinates of these points. Therefore, the set of points in a three-dimensional coordinate system is called a “point cloud”, which is more precisely defined as “thousands of individual measurements in a three-dimensional coordinate system (x, y, z), which a in turn, they compose a three-dimensional model of the registered objects. Although, if the set points have no further processing, they are considered as a very simplified model that operates only visually, because it only consists of unique point entities” [19]. The advantages presented by this system (precision, speed and, generally, software specifically designed for each type of scanner) can sometimes be counteracted by the complexity of the equipment necessary for their management.

Close range photogrammetry (CPR) [20] is considered a rigorous and precise technique. Among its advantages are [21]: (i) the ability to obtain precise and reliable geometries for the reconstruction of three-dimensional objects; (ii) stereoscopic display capacity and (iii) versatility of use of relatively inexpensive instruments. However, as technique it presents some disadvantages, noting that the precision depends on various factors that must be controlled [20], such as: the image scale, image resolution, intersection angles of the images, camera and objective, geometric calibration of the camera, quality of the orientation of the images, image redundancy and the use of markers. According to Rodriguez Navarro, in his article on automated digital photogrammetry, this technique, due to the development of powerful software and digital photography, has been reached as it uses conventional images, carried out in such a way that all parts of the model are visible at least from two different angles [22].

With respect to the auxiliary systems currently used for photogrammetry or short-range 3D scanner, we can distinguish:

- Robotic arms [23]: They automate the process of obtaining data, they are mainly used with the 3D scanner, they position the digitizing equipment making turns and movements around the object.
- Turntables [24,25]: They are widely used in both 3D scanner [26] and photogrammetry [27]. Mainly, its operation is based on the automatic or manual rotation of the platform to prevent the operator from moving around the model, so he can remain static. A system, exclusive for photogrammetry, has also been found in the bibliography, consisting of an automatic rotary table and a manual rotary arm with three predetermined vertical angles and manual focus distance tuning [21].

Currently, 3D scanners are marketed for manual use or mounted on robotic arms. Thus, the experience of the operator is critical when it comes to performing scans correctly. The high cost and low load capacity of the robotic arms, and in addition to that, they also need an auxiliary turntable as well as specific programming per piece or object in order to maintain a fixed distance. Faced with these drawbacks, this work aims to design and build a low-cost semiautomatic system for 3D capture, using the electronic components of a 3D printer as a base and keeping printing capabilities, obtaining a set with the ability to generate 3D models and prints in fused deposition modeling (FDM) too. This system is composed of elements printed from the base 3D printer and commercial components, which facilitate the cloning of the system. The system has the versatility of being able to use cameras of different sizes or different 3D scanners. It can capture images or scan surfaces with controlled movements, reducing the necessity for highly skilled operators. To verify the prototype, this one has already been used to digitize an archaeological piece, corresponding to a plumb ingot found in the “La Tortilla de Linares” foundry [28].

## 2. Materials and Methods

Different studies have been previously carried out for the design of the prototype, which is shown below.

### 2.1. Preliminary Study of Movement

**Figure 1:**
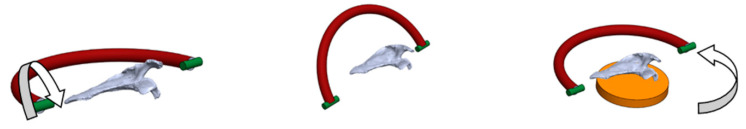
Conceptual Sketches. (Source: self-made).

According to the photogrammetry principles, to encompass the entire sample subject of study, it is necessary to make two 360° turns, one on the X axis and the other on the Y axis, maintaining a constant distance, this strategy will be useful for all those objects whose dimensions are similar to each other and do not exceed those of the turntable. Based on these studies of movement, the need to obtain a spherical movement, whose centre is the centre of gravity of the object to be scanned, has been deducted. Conceptual scheme of the mechanisms must perform the abovementioned spherical movement (Figure 1), where the movements of workpiece and the optical system (either 3D scanner or photo camera) will be combined, i.e., the optical equipment revolved on the X axis and the workpiece on the Y axis at time. To achieve complete digitalization of the piece, it will necessary to turn it over and repeat the process for the part that was occluded. The result is two partial pieces of the scanned object. Then, in the post-processing stage both partial scans will be aligned and it will create a single 3D mesh of the complete object.

### 2.2. 3D Design and Prototyping

**Figure 2:**
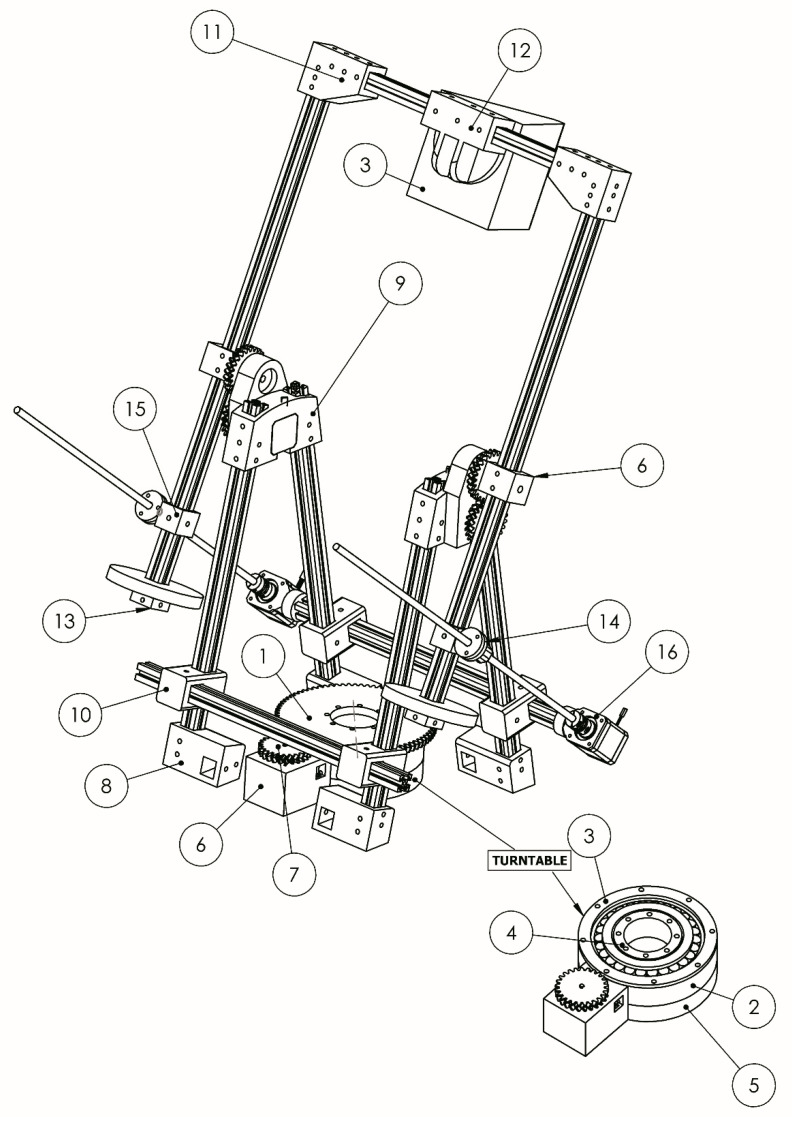
3D Design Model. (Source: self-made).

The description of the different components of the 3D design (Figure 2), can be follow below:

(1) Gear Table: This is a helical gear designed to ensure smooth movements; its double helical shape avoids the axial thrust, a problem that can be found in ‘single’ helical gears. The manufacture of this type of gear is expensive by traditional methods. However, additive manufacturing has not a high cost.
(2) Cradle bearing table: A cradle is done to accommodate the turntable bearing. The cradle is designed with the same inclination as the bearing, creating a perfect fit.
(3) Bearing closure table (Outside diameter): This upper closure prevents that the bearing can get out.
(4) Bearing closure table (Inner diameter): It is the lower close of the bearing, allowing their movement, but avoiding ejecting.
(5) Table supplement: A supplement of the turntable is designed to adjust to the measurement of the chosen engine.
(6) Engine supplement: This is a piece of engine adjustment used to make the gear pinion-motor spin with the gear table.
(7) Gear Engine: The pinion of the turntable, designed in conjunction with the gear table, is double helical so as to overcome the problem of axial thrust helical single gear.
(8) Support structure: It performs its function as it supports the soil which stiffens the structure, allowing the fixing of aluminium profiles.
(9) Top Union: The connection piece and the rotation centre are responsible for attaching the rigid structure with the moving part.
(10) Union structures: They consist of the joints between the aluminium profiles, to form the backbone of the machine and to stiffen.
(11) Upper elbow: Higher elbows have a function of distance adjustment for different models of cameras and 3D scanner, anchoring the top post at the same time.
(12) Support 3D scanner/camera: It is a top bracket to support the 3D camera or 3D scanner.
(13) Counterweights stops: The adjustable stops serve to fix the weights balancing structure according to the mounted device.
(14) Head electric piston: This allows the passage of the rods and it is articulated, achieving the simulation of the function of a piston.
(15) Rotating rots piston unions: It is the coupling between the rotating bars and the electric piston head.
(16) Lower joint piston: This is the coupling between the fixed structure and the threaded piston electric barillas.

Once the design has been obtained, manufacturing the system will be made fast by prototyping. The possibility of having low-cost models is a relatively new possibility and it has occurred thanks to the release of patents and low-cost electronic systems [29,30]. Although 3D design software engineering reduces errors in previous designs, prototypes are still indispensable for testing and final adjustments. That is the reason why, after performing the virtual design, the prototype is manufactured using additive 3D printing FDM technology, with the intention of presenting a functional design. Different software applications (Autodesk Inventor [31]; Netfabb Basic 2012 [32]; Cura 2016 [33]; Repetier-Host 2016 [34]) has been used for 3D design. The 3D FDM printer and the material used have been BCN3D+ [35] and ABS filament (1 kg), respectively.

The structural set enables the adjustment of the different camera or 3D scanner models, modifying the elements (6), (8), (10), (11), (13) and (15) shown in Figure 2, allowing the variation of the workspace, which can be easily adapted by replacing the aluminum profiles and modifying the initial system counterweights.

The combination of the gear, the turntable and the supplement, (7), (1) and (5) in Figure 2, allows the system to support up to 60 kg of weight. The twist is unlimited and the helical gear system can perform smooth movements. The bearing selected to withstand a wide variety of loads is an NTN 30216U bearing [36].

The designed prototype is a flexible system that allows changing the working dimensions and 3D capturing device. As a summary, the main capabilities of the system are:

- Maximum working dimensions 400 mm × 400 mm × 500 mm. Customizable mean replacing aluminum profiles.
- Turntable with 60 kg capacity and unlimited horizontal rotation.
- Vertical angle of rotation (pitch) of 120°. As noted above, the object must be flipped if the geometry of the entire object is to be captured.

The set can reach a rotation (pitch) of up to 120°, through the lower joint piston, the head electric piston and the top union, (6), (14) and (16) in Figure 2, allowing to record parts located in the lower side of the pieces, reducing to a minimum the changes in their position.

Once the system has been configured for the corresponding 3D scanner or camera model, the movements carried out are programmed in G-code, and taking into account the specific characteristics of each system used. In general, for 3D scanners, G-code is used with a continuous movement system, without stopping, simulating the speeds recommended by the manufacturers.

The strategy followed for photogrammetry is different, to avoid dependence on high-speed cameras and lenses, due to their high cost. The G-code file contains the orders to reach the desired capture positions through the movements and, once achieved, stops the equipment and proceeds with the image capture, resuming the movements again to the next position.

### 2.3. Structural Components

**Figure 3:**
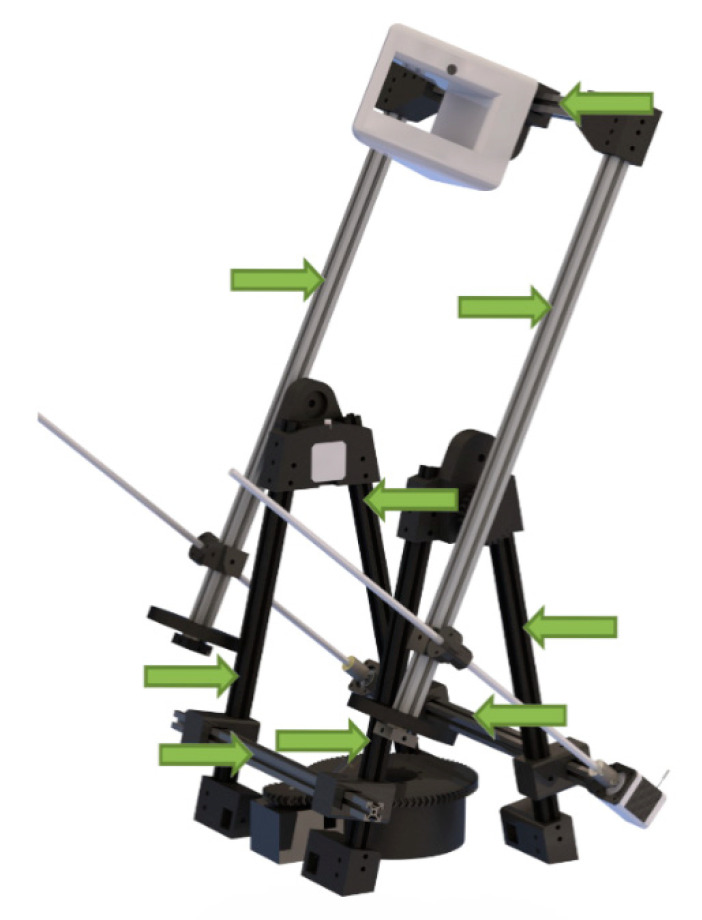
Structural elements. (Source: self-made).

Structural components (Figure 3) have been chosen according to the assumptions raised from initial sketches and should be low-cost and easy to find on the market. Extruded aluminum profiles are commonly used in industry for manufacturing machines, supports, fences, among others. Due to their flexibility, high variability and lack of machining, assembly costs are reduced. The main bearing involved in turning the table has been selected to withstand the high radial and axial load [36].

Coupling steel binding is a flexible connection between the stepping motor and threaded rod, allowing slight movements while preventing breakage and vibration. Auxiliary elements such as threaded rods, bolts and balances can be found.

### 2.4. Electronic Components

**Figure 4:**
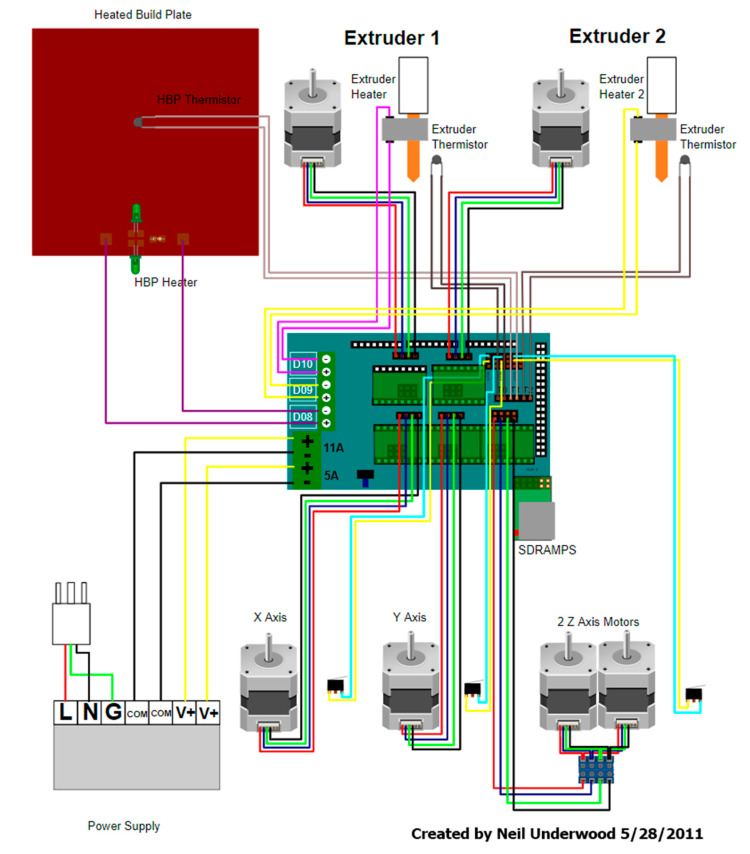
3D printer Scheme. (Source: [37]).

**Figure 5:**
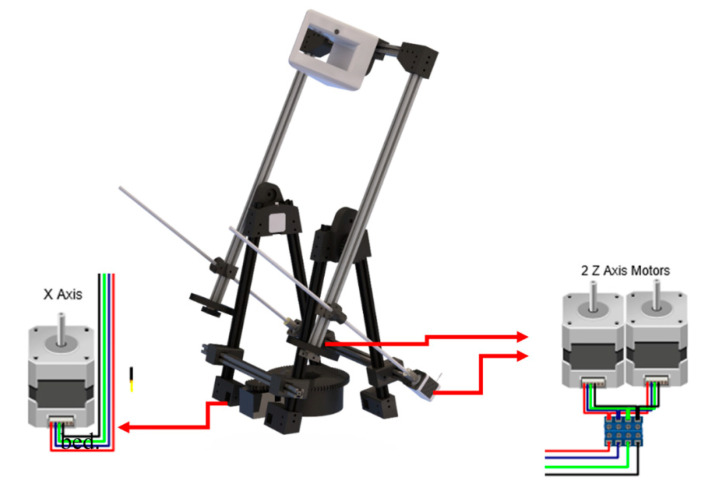
Motors Location. (Source: self-made).

In the case of electronic components, the use of basic open 3D printer software and Arduino-based hardware firmware has been selected. In Figure 4, the outline of these printers is shown. The list of electronic components used in the prototype and their function is found below:

- Arduino Mega 2560: It is a printed circuit board with a microcontroller with digital and analog input/output.
- Ramps v1.4 Arduino expansion board: It is designed to accommodate all the components, inputs and outputs of the 3D printers.
- Pololu Set Purple: Drivers are stepper motors.
- Stepper motors Nema 17, 1.8° pitch angle (200 steps per revolution): Two motors in the corresponding pin of the Z axis are responsible for the optical equipment rotation, and in the X axis, one is responsible for the rotation of the movable table (Figure 5).
- Power Supply of 12 V and 30 A: It is responsible for feeding the system.

Compared to the Prusa I3 3D printer, which has been taken as a reference, our prototype initially, uses only three motors during 3D scanning work, instead of five when the system works as a 3D printer, since it does not use temperature or hot bed probes. The use as 3D scanning device or 3D printer is programing via software.

To take advantage of these printers, a firmware is required to modify the programming code. This firmware is designed to carry out controls of all the components and to adapt to the speeds necessary to make movements in the Y and X axes and to rotate the threaded rods according to the units that we need to increase the value of the Z axis. In summary, the changes needed in the firmware are:

- Elimination of temperature controls, since if a probe is not detected in the printer, it would return an error and do not load movements.
- Change the rotation speed of the motors and their conversion to real angles.
- The spindle motor table stops are removed.
- The stops on the spindle motor of the optical system was modified too.

To achieve the mixed 3D printing and 3D digitizing system to work in the same device, modifications made to the firmware can be optional and allow the user to select each system. The cost increase of the inclusion of the scanning system in a Prusa I3 3D printer is reduced to:

- Three Nema 17 stepper motors (40 €)
- Aluminum profiles (60 €)
- ABS filaments (30 €)
- Bearing (25 €)
- Small mechanical and electrical auxiliary components (20 €)

### 2.5. 3D Capture Equipment

Within the conceptual and imposed premises of the prototype design, the ability to adapt different 3D scanner or camera models was basic, avoiding dependence on certain models.

For the selection of the equipment we have been based on the following conditions that would justify the design of the prototype used in each technology.

- 3D scanner
  The scanner selection should serve a dual role:
  1. Verify the results obtained by the photogrammetric system, so a scanner with a precision below 0.1 mm, should be used, for the objectives pursued.
  2. Using a 3D scanner is not an easy task and requires an experienced operator, where multiple operators will not scan in the same way. Being able to use it automatically and always the same way is a very ambitious goal that the system is capable of achieving.

In summary, the main condition will be to automatically use a scanner with an accuracy below 0.1 mm.

- Photogrammetry

Use of mid-range price DSRL cameras with zoom lenses. Although these lenses are not the most suitable for photogrammetry, they incorporate high versatility as they are commonly used in archaeology, medicine and by occasional photographers. Professional cameras with high quality fixed lenses have been discarded, because mounting them in the system would not allow the system to be considered low cost. Although they can be mounted effortlessly, obtaining more accuracy and professional results.

A fixed lens has is priced similar to or even higher than zoom lens, but a multitude of fixed lenses will have to be purchased to obtain the same scan range as a zoom lens, increasing the cost of the system disproportionately.

While it is true that a zoom lens must always be calibrated before each scan session at unique focal length, technically the fixed lenses must also be calibrated before each data acquisition, since temperature influences aberrations, this is an often forgotten basic photogrammetry concept.

**Figure 6:**
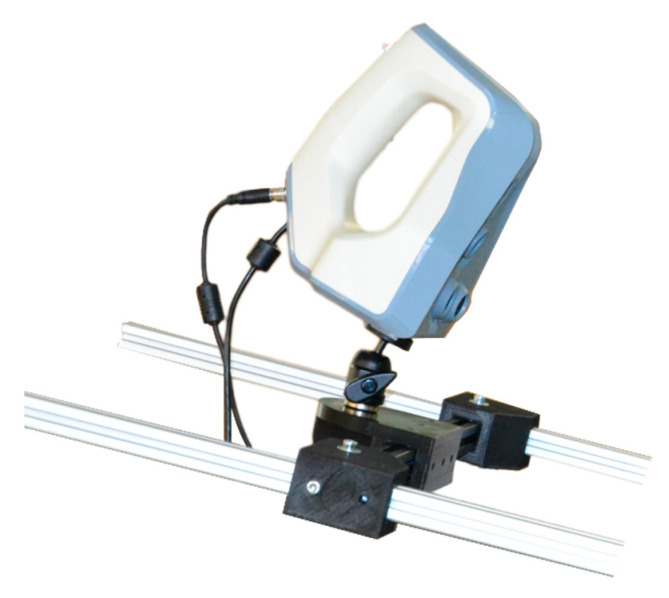
Spider Artec 3D scanner. (Source: Self-made).

In summary, for this work, the main condition in photogrammetry will be to use a camera with a commercial lens and priced below 600 €. For field testing the equipment described below will be used:

(1) Spider Artec 3D Scanner [38] (Figure 6) whose specifications are shown in Table S1 in the Supplementary Material.

The Spider Artec is a 3D scanner with a precision of 0.05 mm and has a restricted working distance (range between 170–340 mm), causing common problems of loss of references due to sudden movements, being a scanner only suitable for very experienced operators. The developed prototype has made the process automated with gentle movements and can be used by any operator with basic training.

**Figure 7:**
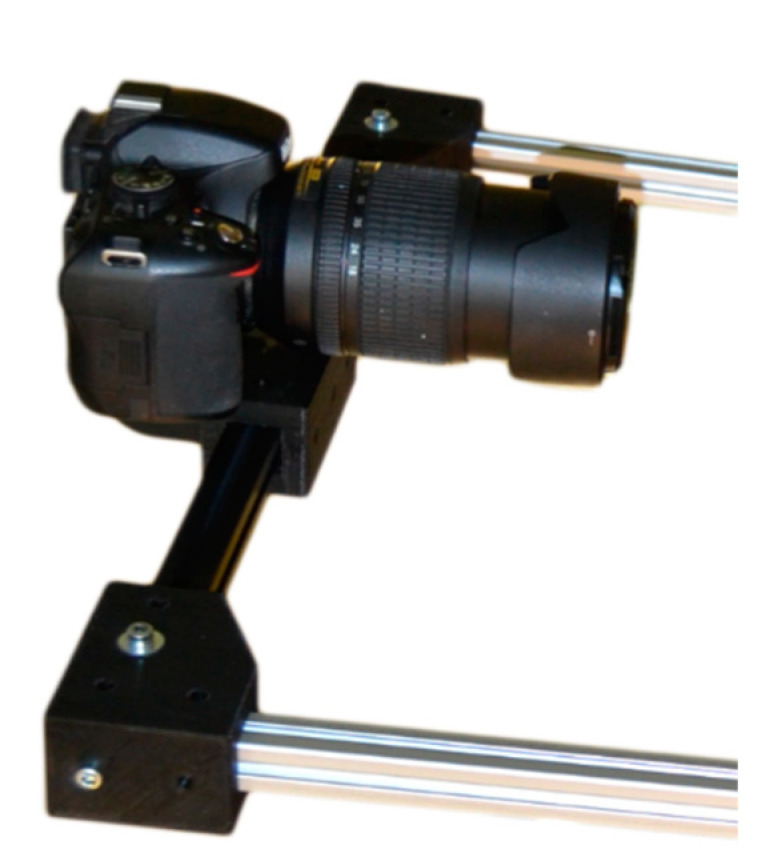
Nikon Camera. (Source: Self-made).

(2) Nikon 5100 SLR camera equipped with a Nikon AF-S 18–105 mm 1: 3.5–5.6 G ED [39] (Figure 7). This is a mid-price range camera with a basic and widely used lens, which allows one to validate the photogrammetric system, bringing the possibility of 3D digitization closer in many sectors without a large investment. 

### 2.6. Data Collection

Once all components are assembled, Arduino programming is modified, and movements are verified; final checks are performed with the 3D scanner and camera.

To control the movements of the equipment, machine code (G-Code) is used, which is the same code that laminators create when generating files for 3D printing, these can be saved to be used according to different customization setups (preset).

The data generated by the 3D scanner is processed directly by the Artec Studio software since the scanner remains connected to the computer equipment.

The data generated by the camera is stored on the SD card, and can be accessed from the USB cable connected to the computer. To perform three-dimensional capture, several methods are used:

#### 2.6.1. Scanning Using Prototype and Artec Spider Scanner

**Figure 8:**
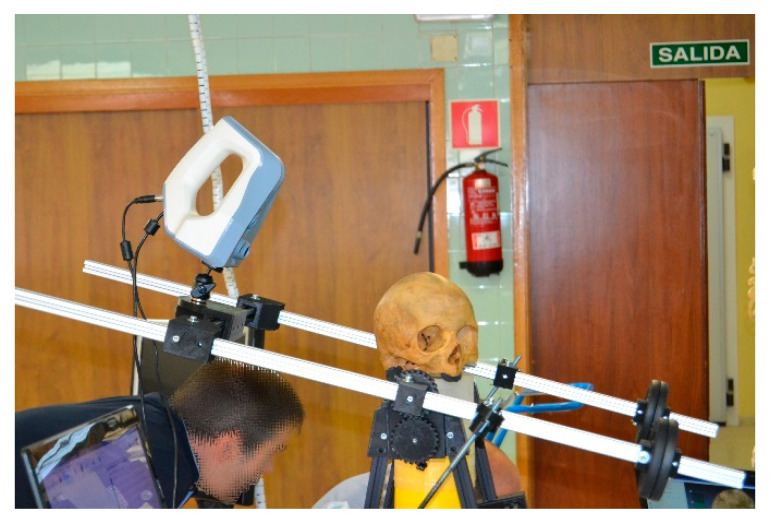
Prototype with mounted scanner (Source: self-made).

The scanner will be placed in the prototype through a tripod ball head mount and synchronized with the turntable (Figure 8).

The generation of 3D model was made with Artec Studio software after scanning. The scanning process characteristics are:

- Scanner warm-up time: 30 min, according to manufacturer’s specifications to ensure good accuracy.
- Distance: the scanner distance to object was set in 260 mm. The spherical movement is the responsible for keeping the maximum and minimum values within the scanner range.
- Scanning time: 13 min.

#### 2.6.2. Scanning Using Prototype and DSRL Camera

**Figure 9:**
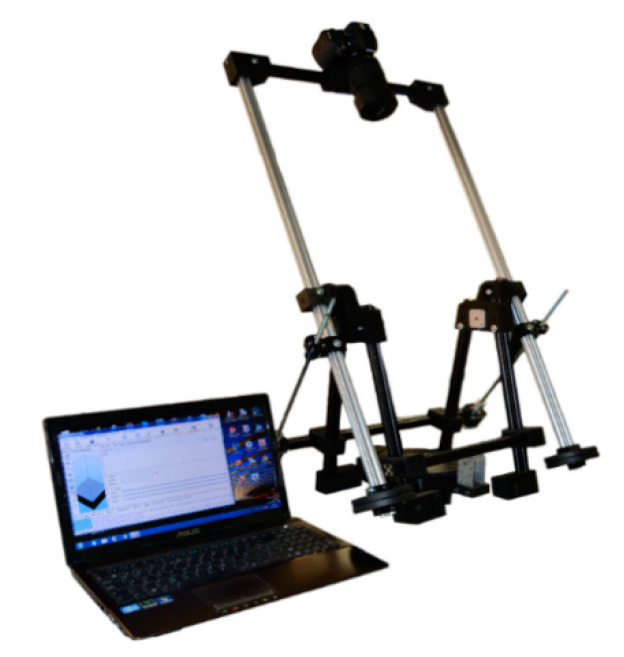
Prototype with camera mounted (Source: self-made).

In the photo shoot, the prototype has been used for generating 3D models after photogrammetric digital image processing (Figure 9).

The scanning characteristics are:

- Camera heating time: the camera does no need heating time, but it need prior calibration for the lens and focal length chosen for the job The Agisoft Lens software [40] is used to calibrate the camera, taking into account that the calibration is only valid for the chosen focal length. The process is described below

1. A calibration chessboard pattern is placed on the base of the system (turntable platform).
2. The camera is placed in the system and 21 photographs are taken (three are the minimum for calibration). To obtain the images, the indications of the software will be taken into account:
  ▪ Dazzling light sources will be avoided.
  ▪ The photo area should cover the entire calibration pattern.
  ▪ Maintaining the working focal length

These images are made with different angles. This process is carried out automatically by loading the G-code file “calibration”, which has been generated for the system. These automated shots with different angles ensure a calibration with very stable and reproducible results.

The G-code file starts by moving the turntable at 15° angles and capturing the pattern, keeping the camera angle fixed. Once the 90° rotation of the table is completed, the arm vertical angle is increased by 30°, proceeding again to rotate the table and take the captures. It ends with the arm at a vertical angle of 60° and the last captures are made by moving the rotary table.

- Distance: The distance is set in 450 mm. The spherical movement is responsible for keeping within the maximum and minimum field of view of the target values.
- Scanning time: 10 min.
- Retrieved file: 180 pictures with 4928 × 3264 pixel and .jpg format occupying 756 MB. Although it is recommended, they have not been obtained in raw form (raw camera data) to speed up working times.
- 60 mm focal length selection: with this distance the camera is calibrated.
- ISO-200
- Shutter speed of 1/60 s.
- 80% overlap of photos.

### 2.7. Edition of 3D Meshes

**Figure 10:**
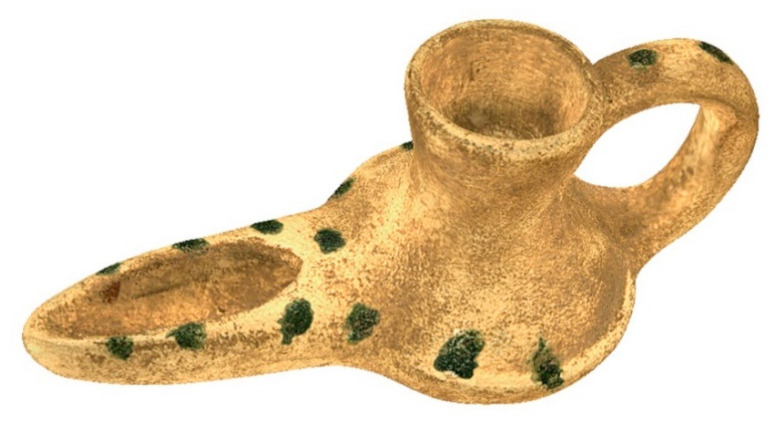
Oil Lamp object of study. (Source: self-made).

Once field data have been obtained, 3D models of scanned pieces are generated. The selected piece object of 3D scanning is a oil lamp (Figure 10), used for lighting, whose use has been recorded since the 10th century B.C., not previously digitized. This archaeological piece has been chosen for the complex shapes it presents and for the interior gaps. This work is done with specific software, depending on the equipment used for 3D data capture.

#### 2.7.1. Artec Spider Scanner with Software Artec Studio 11

This software [41] controls the scanner during the data collection; it is employed later for post processing and for getting the 3D mesh, using a multitude of specific algorithms for obtaining high-quality models, with an accuracy of 0.05 mm, as indicated above.

The software has several modes of operation. The “manual operating mode” allows the operator to decide on the parameters and obtaining greater precision compared to the “automatic mode”, while in the “Autopilot For beginners mode”, the software decides and simplifies the obtained models, using different automatic algorithms. For best results, Artec recommends “manual mode”, which is why it has been used in our study, providing precision and reliability.

The scanning methodology is based on:

(1) Connect the scanner to power and the control computer.
(2) Place the scanner in its working range, this range can be checked in the scanner window preview.
(3) After 30 min of warm-up of the scanner, speed tests are carried out to program the G-code file, determining the range of speeds allowed by the scanner.
(4) Loading the G-code file in the system with the speeds established in the previous point.
(5) Choosing the scanner configuration for data acquisition.

As indicated by the manufacturer, it is advisable to scan all sides of the object at once, slowly moving the scanner around it, a very complicated option without the automatic system. In summary, the data capture with the prototype is done automatically, while the data processing with the software will be in “manual mode”.

Once the scan data has been obtained, the results will be processed by:

- Scans alignment
- Registration: Through algorithms, the positioning of the scans obtained is optimized.
- Eliminate Noise
- Fusion: Create a single surface model from the multiple surfaces obtained during the scan.
- Postprocessing

Defects can also be corrected with a smoothing and a photo-realistic texture will be applied. This procedure allows scanning of objects, adjusting very precisely the rotational speed of the rotary table and the rotation of the scanner. The G-code files used are stored for each scanner model, thus obtaining a quick system configuration, in which it will only be necessary to calibrate the speed when using a new 3D scanner.

One of the keys to selecting the Artec Spider scanner has been precisely the sensitivity it shows against small sudden movements of the operators, the speed settings on the Artec scanner have not presented any problem, obtaining the same results as a specialized operator. Thus ensuring a wide range of scanners that can be used with the designed system.

#### 2.7.2. Photogrammetry with DSRL Camera

**Figure 11:**
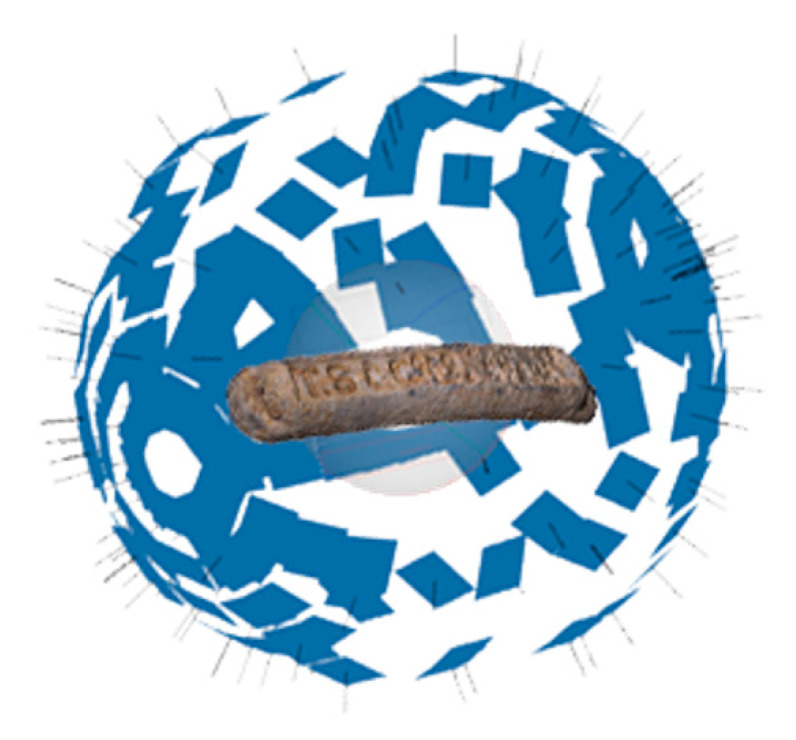
Sphere obtained from the positioning of the photographs. (Source: self-made).

For generating 3D models from photographs, the Agisoft PhotoScan software (currently renamed as Metashape) [42] was used. Once the images are obtained, they are oriented, forming an almost perfect sphere generated by positioning the photographs, once aligned (Figure 11). This area would not be easily achieved without the automated process of our system. Manually, good results could be obtained depending on the skill of the operator; besides being quite complicated repetitiveness in their quality.

Below is the working process in more detail:

- Upload photos: Uploading the photos consists of selecting the folder where the photographs taken are located. This folder must contain only the correct photos.
- Selection of calibration and reference points: Markers are used on the platform to scale the system; these markers are automatically recognized by the software. The coordinates of these markers have been taken with a Hexagon Romer arm with a precision of 0.03 mm.
- Alignment: To align the images, the Accuracy parameter will be selected as high to work with the original image resolution. This setting will require more processing time but will allow us better precision. The “key point limit” and “tie point limit” parameters, are kept at their default value.
- Creation of the point cloud: To generate the dense point cloud, the medium level parameter is selected because a higher quality requires a higher processing time. Once the mesh is generated, noisy points are fixed and cleaned manually.
- Mesh creation

This step allows generating a 3D mesh, triangulating the points of the dense cloud. Given the size of the object and its morphology, the options chosen will be:

Surface type: Arbitrary (3D)
Source data: Dense cloud
Face count: High
Interpolation: Enabled (default)
Point classes: All

With these options, the 3D mesh is obtained with the greatest possible detail.

- Texturing

Is the last step in the process and, although it has no effect on a dimensional level, it get a much-appreciated photo-realistic display. The selected options are:

Mapping mode: Generic.
Blending mode: Mosaic (default)
Texture size/count: 2048 × 1

Using the prototype with continuous capturing, no stops programmed, requires high shutter speeds in the camera and a good lighting system. The speed of the system turns in the G-code file is coordinated with the shutter speed to obtain photographs in focus, without trepidation, and with the detail necessary for recognition of points of interest. Since any movement, those associated with the direction of translation and rotation can cause more blurry images [43], it is necessary that the shutter speed be determined by the speed of the turntable and vertical movement, as well as the focal length of the lens (reciprocity principle).

## 3. Results and Discussion

### 3.1. Comparing 3D Meshes

**Figure 12:**
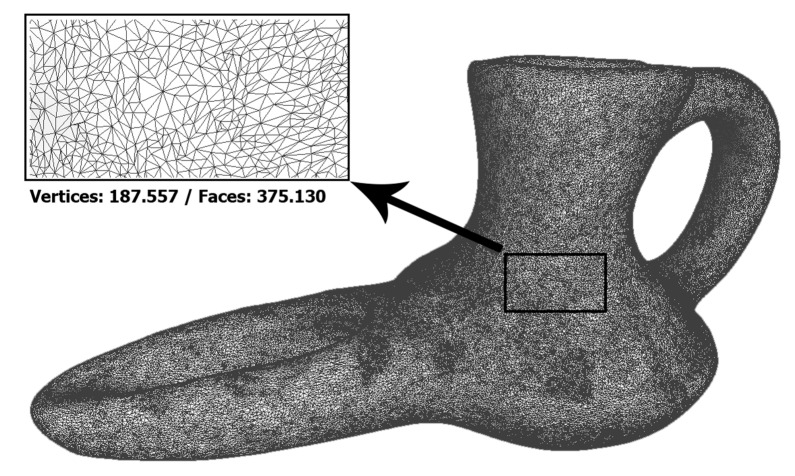
Meshes obtained by Artec Spider. (Source: self-made).

**Figure 13:**
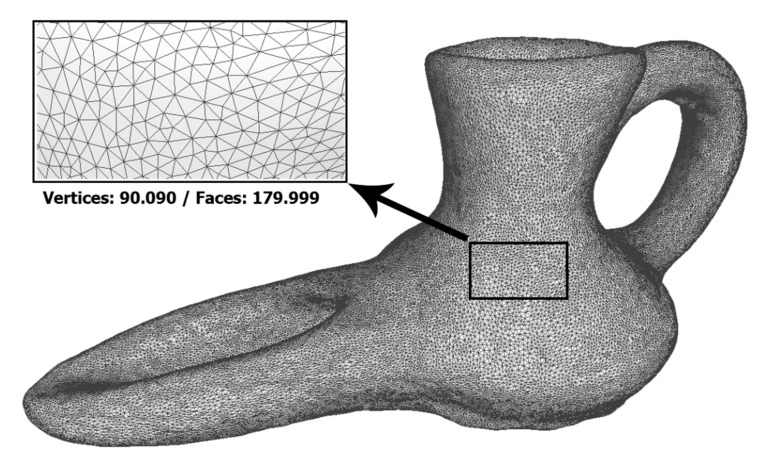
Meshed obtained by photogrammetry. (Source: self-made).

The meshes obtained from the two systems are shown below (Figure 12 and Figure 13), as well as their characteristics. In them it can be seen that the resolution of the mesh from the 3D scan is approximately double that obtained by photogrammetry. This difference would be practically equalized if the photogrammetric dense point cloud had been generated with high quality values. However, this increase in the number of polygons does not provide relevant information for the object of study and, on the contrary, would generate an extra cost in processing times.

**Figure 14:**
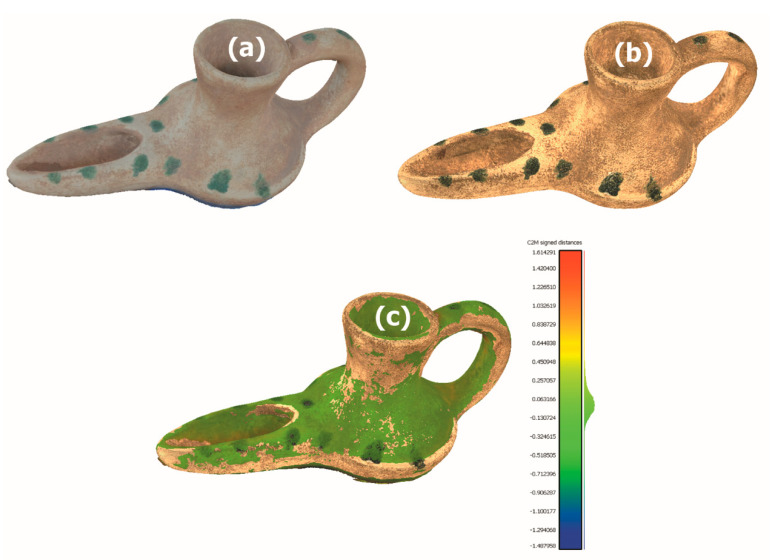
(**a**) Meshes Captures with photogrammetry (**b**) Meshes Captures with scanner Artec Spider (**c**) Corporate image with colour on mesh. (Source: self-made).

To check the accuracy of the results, the Open Source software Cloud Compare is used [44]. The 3D mesh obtained with the 3D scanner is taken as a reference and compared, using this software, with the one obtained using photogrammetry. Once computed the distances between homologous points, the overlap of the meshes obtained by photogrammetry and 3D is generated, creating a colour scale pattern according to the deviation with the corresponding mesh obtained by scanning (Figure 14c).

In Figure 14 can be observed the both meshes of the selected piece, obtained with photogrammetry (Figure 14a) and with the 3D scanner in manual mode (Figure 14b), being the points of the interior gaps where the greatest deviation is shown.

Figure 14c is obtained from the superposition (best-fitting method) of the meshes from scanning and photogrammetry, marking a limit of deviation of ±0.2 mm, all the points that are below this limit will appear in green. It can be seen that the area below ±0.2 mm is very representative.

**Figure 15:**
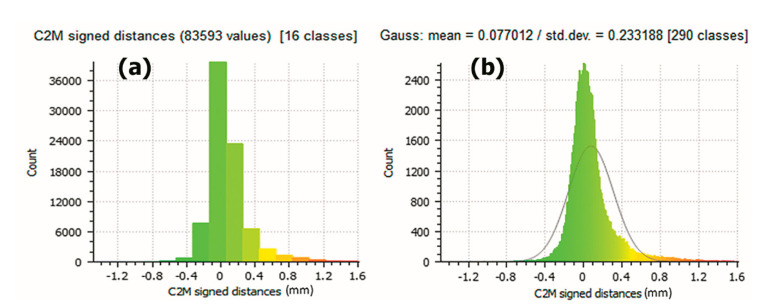
Charts for the Histogram and distribution generated by the Software Cloud Compare. (Source: self-made).

In Figure 15 clusters and distribution differences between the vertices of the mesh obtained by photogrammetry and the mesh obtained with the 3D scanner can be observed. It can be seen that graphics tends to zero difference, showing the efficiency of the system. In Figure 15a the graph groups the points according to the coincidence of values between 16 groups, these represent the trend of the precision of the values. In Figure 15b the Gaussian bell is represented, showing how the probability of the values is distributed, noting that the most common values appear in the center of the bell and the less frequent, at the ends.

**Figure 16:**
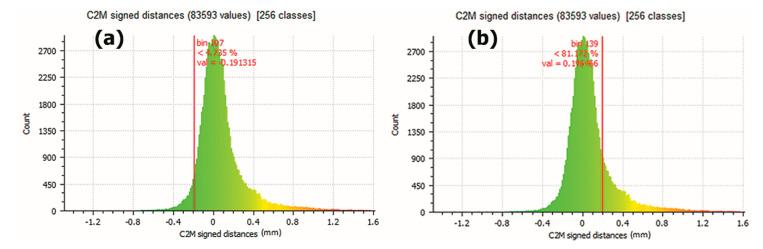
(**a**) Results showing that 18.828% of the points have a larger difference than 0.2 mm. (**b**) Results showing that 4.735% of the points have a smaller difference than −0.2 mm. (Source: self-made).

If a limit of ±0.2 mm is set in the error value, it is obtained that 18.828% of the points have a difference with each other larger than 0.2 mm (Figure 16a) and 4.735% of them have a difference under −0.2mm (Figure 16b). In the central part of the graphic, the 76.437% of 83,593 points are included in a smaller difference than ±0.2 mm.

**Figure 17:**
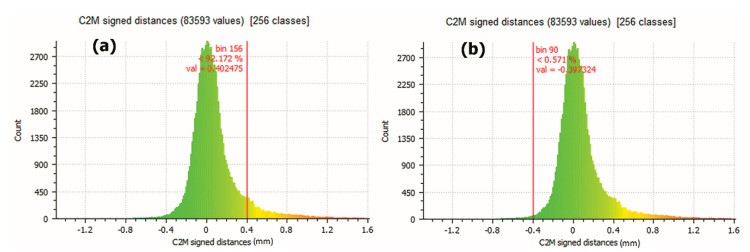
(**a**) Results showing that 7.828% dots have a larger difference than 0.4 mm (**b**) Results showing that 0.571% of the points have a smaller difference than −0.4 mm. (Source: self-made).

If the limit is increased to ±0.4 mm, the 7.828% of the points have a difference with each other greater than 0.4 mm (Figure 17a) and 0.571% of them have a minor difference of −0.4 mm (Figure 17b). In the central part of the graphic, the 91.601% of 83,593 points are included in a smaller difference than ±0.4 mm.

As summary, if a limit of ±0.2 mm is marked (Figure 16), the coincidence of the points represents 76.437% between the points of the two meshes. This percentage of coincidence rises to 91.601% if a limit of ±0.4 mm is established (Figure 17).

**Figure 18:**
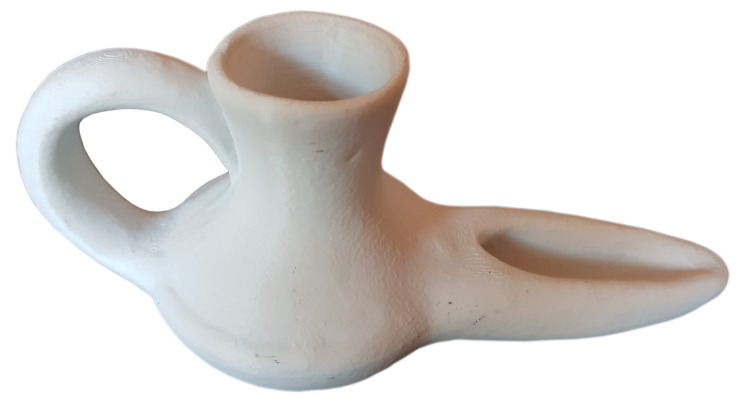
Printed model obtained by photogrammetry (Source: self-made).

Figure 18 shows the result of the 3D printed model obtained from photogrammetry 3D scanning, the printing has been made with the same device working as 3D printer that controls the designed prototype, validating the system. The printing has been made with ABS filament and with a layer height of 0.1 mm.

The contributions that the designed device generates are:

(1) Common Contributions to Photogrammetry and 3D Scanning.

- The design of the device has been developed specifically to carry out 3D scanning or photogrammetry tasks, being able to compare the results of the robotic arms, reducing their cost by more than 40 times.
- Very flexible adaptation that allows using of different 3D scanners or cameras for photogrammetry.
- Since it takes advantage of the electronics of open source printers it can work as a dual system of digitizing and 3D printing.
- The ability to adapt to different equipment together with the possibility of modifying the distance to the model allows digitizing with the current system dimensions ranging from 10 to 500 mm, allowing increased resolution and precision in the necessary models in combination with the digitized system.
- Automation of the process with the possibility of generating predetermined programs (custom presets) for beginning users, allowing to reduce the learning curve for both, 3D scanning and photogrammetry.
- The system form a virtual sphere for data capture, which avoids the appearance of areas without information, reducing the possibility of missing data.
- Scanning and photogrammetry capabilities without losing the system’s printing capabilities. The system can work in conjunction with an FDM 3D printer, obtaining printable digital models in the system.

(2) Specific contributions for Close Range 3D scanning

- Total control over the rotation speeds that allow an ideal adjustment to the scanner model used.
- In manual scanning processes the operator is one of the most important variables in the final results. The automation of the scanning process allows inexperienced operators to obtain the same results as an advanced operator, managing to compete with professional robotic systems [15].

(3) Specific contributions to close range photogrammetry

The designed prototype improves the control of the properties that must be considered in photogrammetry, as indicated in the introduction, which are described below:

- The image scale: The prototype allows selecting the maximum camera-object distance, checking that the precision is sufficient for the job.
- Thanks to the flexibility of the prototype, you can choose the camera with the appropriate resolution for the desired precision.
- The intersection angles of the images are controlled with accuracies of less than 1°, allowing better planning according to the type of piece and improving automation and angular control of semi-manual devices with fixed angles [40] that present problems depending on the piece of study.
- The prototype helps and automates the geometric calibration of the different cameras that are coupled to the system.
- The image orientation quality is evident in the alignment spheres of the system.
- With the programming in G-code the redundancy of images is achieved as desired.
- The use of markers in areas of the table helps and improves the manual process.
- The main disadvantages of our system are:
- It is necessary to modify the position of the piece to capture the occluded areas; even so the system reduces 3D scanning to two positions per piece, improving traditional systems.
- The scanning distance once set does not vary during data collection. Occurs the possibility of the implementation of a proximity sensor that modifies the distance adapting it to the geometry of the object, managing to maintain the optimal distance at any time.

## 4. Conclusions

From the preparation of this work it can be concluded that after studying preliminary ideas and sketches, a virtual design has been made and validated, affording a functional, robust and adaptable prototype for 3D scanning with any type of camera/3D scanner combination. This can be seen by comparing the results obtained by the photogrammetry and the 3D scanner, which demonstrate that the use of photogrammetry is feasible, which reduces the costs that this entails in the acquisition of a 3D scanner. It illustrates that the positions obtained with the use of photogrammetry generate regular areas that are practically impossible to reach manually. Therefore, the degree of specialization of the personnel that captures the data is transitively reduced, resulting in uniform and reproducible results.

In the design of the equipment, maintenance of the original functionality of the 3D printer has prevailed, creating a team capable of creating 3D models and printing them. Since the base of the system is an open source FDM printer, the set developed has an exponential expansion potential.

For applications that require greater precision, when a 0.5 mm deviation is unacceptable, the number of positions used to generate 3D models using photogrammetry can be increased through a simple programming modification. In addition, the disposition of the different movements will be saved at any time to select the most appropriate one. The use of quality objectives and fixed focal length have fewer aberrations as they have fewer construction elements and these are of better optical quality. The possibility of mounting several cameras in the system for double/triple positions can also be considered.

As future perspectives that have emerged in the development of this work and that were not expected in the initial objectives, it is necessary to emphasize the ability to digitize pieces in order to create 3D virtual libraries, with applications in various fields, such as forensics, museums, archaeological studies, among others. In the future, these libraries could be shared between institutions and automated studies.

On an industrial level this system can be used for capturing and reproducing industrial spare parts, quality control in the industry, since it can capture parts to compare them with the CAD models designed and verifying tolerances or identifying manufacturing errors.

At the level of consumption, this equipment can be implemented as a parallel device in 3D printers, since in recent years, different three-dimensional capture equipment has proliferated at the user level, and this equipment has great versatility and quality in the 3D meshes obtained.

## Data Availability

Not applicable.

## Supplementary Materials

The following are available online at https://www.mdpi.com/article/10.3390/s21082580/s1, Table S1: 3D scanner Spider Artec, Table S2: Nikon camera characteristics.

## References

[1] Pichler G. (2015). 3D Laser scanning—Application in plant engineering and construction. AT Miner. Process..

[2] Peck L.R., Cheng M.L. (2016). The accuracy of an optimized, close-range photogrammetry practical method for vehicular modeling. SAE Int. J. Transp. Saf..

[3] Barrile V., Meduri G., Bilotta G. Laser scanner surveying techniques aiming to the study and the spreading of recent architectural structures. Proceedings of the 2nd WSEAS International Conference on Engineering Mechanics, Structures and Engineering Geology.

[4] Xiong X., Adan A., Akinci B., Huber D. (2013). Automatic creation of semantically rich 3D building models from laser scanner data. Automat. Constr..

[5] Achille C., Adami A., Chiarini S., Cremonesi S., Fassi F., Fregonese L., Taffurelli L. (2015). UAV-based technologies for photogrammetry and integrated architectural strategies for the Applications—Methodological after-quake survey of the vertical structures in Mantova (Italy). Sensors.

[6] Dubbini M., Curzio L.I., Campedelli A. (2016). Digital elevation models from unmanned aerial vehicle surveys for archaeological interpretation of terrain Anomalies: Case study of the Roman castrum of Burnum (Croatia). J. Archaeol. Sci. Rep..

[7] Guidi G., Russo M., Angheleddu D., Zolese P. A virtual connection Between past and present: The digital revival of Chams Architecture (Vietnam). Proceedings of the 18th International Conference on Virtual Systems and Multimedia.

[8] Yamafune K., Torres R., Castro F. (2017). Multi-Image Photogrammetry to Record and Reconstruct Underwater Shipwreck Sites. J. Archaeol. Method Theory.

[9] Haleem A., Javaid M. (2019). 3D scanning applications in medical field: A literature—Based review. Clin. Epidemiol. Global Health.

[10] Gür Y. (2015). Additive manufacturing of anatomical models from scan data Computed Tomography. Mol. Cell. Biomech..

[11] Lussy P., Lussu P., Marini E. (2020). Ultra-close range digital photogrammetry in skeletal anthropology: A systematic review. PLoS ONE.

[12] Caravaca G., Le Mouélic S., Mangold N. (2020). 3D digital outcrop model reconstruction of the Kimberley outcrop (Gale crater, Mars) and its integration into Virtual Reality for simulated geological analysis. Planet. Space Sci..

[13] Deli R., Di Gioia E., Galantucci L.M., Percoco G. (2010). Automated landmark extraction for orthodontic measurement of faces using the 3-camera photogrammetry methodology. J. Craniofacial Surg..

[14] Remondino F., Guarnieri A., Vettore A. (2004). 3D modeling of Close-Range Objects: Photogrammetry or Laser Scanning. Proc. SPIE.

[15] Galantucci L.M., Guerra M.G., Lavecchia F. (2018). Photogrammetry Applied to Small and Micro Scaled Objects: A Review.

[16] Guerra M.G., Lavecchia F., Maggipinto G., Galantucci L.M., Longo G.A. (2019). Measuring techniques suitable for verification and repairing of industrial components: A comparison among optical systems. CIRP J. Manuf. Sci. Technol..

[17] Sims-Waterhouse D., Piano S., Leach R. (2017). Verification of micro-scale photogrammetry for smooth three-dimensional object measurement. Meas. Sci. Technol..

[18] Kwan Y. (2016). Laser Scanning, Theory and Applications.

[19] Mañana P., Rodríguez S., Blanco R. (2008). Una experiencia en la aplicación del Láser Escáner 3D a los procesos de documentación y análisis del Patrimonio Construido: Su aplicación a Santa Eulalia de Bóveda (Lugo) y San Fiz de Solovio (Santiago de Compostela). Arqueol. Arquit..

[20] Luhmann T., Robson S., Kyle S., Boehm J. (2013). Close Range Photogrammetry and 3D Imaging.

[21] Galantucci L.M., Pesce M., Lavecchia F. (2015). A powerful scanning methodology for 3D measurements of small parts with complex surfaces and sub millimeter-sized features, based on close range photogrammetry. Precis. Eng..

[22] Rodríguez N.P. (2012). Digital photogrammetry systems based on versus the active 3D sensors. Expresión Gráfica Arquit..

[23] Artec 3D. https://www.artec3d.com/portable-3d-scanners/robotic-scan.

[24] Titenia 360°. https://titania360.com.ar/#modelos.

[25] 3D Work. https://3dwork.io/scanner-3d-con-arduino-y-fotogrametria/.

[26] BQ. https://www.bq.com/es/support/ciclop/support-sheet.

[27] Matter and Form. https://matterandform.net/scanner.

[28] González-Merino R., Fraile A.D., Pérez J.A., Sánchez-López E. (2017). Validation of photogrammetry techniques performed on two lead ingots assigned to Linares Historical Heritage. Proc. Manuf..

[29] Arduino (2016). Arduino (GPL Software). Versión 1.6.12. https://www.arduino.cc/.

[30] Raspberry Pi (Software). www.raspberrypi.org.

[31] Autodesk Inventor (Software). http://www.autodesk.es/products/inventor/overview.

[32] Netfabb (2012). Netfabb Basic. (Software). http://www.autodesk.com/products/netfabb/overview.

[33] Cura (GPLsoftware) (2016). Version 2.3. https://ultimaker.com/en/products/cura-software.

[34] Repetier (2016). Repetier-Host (software). Version 1.6.2. https://www.repetier.com/.

[35] BCN3D Technologies. https://www.bcn3d.com/es/.

[36] NTN-SNR. https://eshop.ntn-snr.com/es.

[37] RepRap. http://www.reprap.org/wiki/File:Rampswire14.svg.

[38] Artec 3D. www.artec3d.com.

[39] Nikon. www.nikon.es.

[40] Agisoft Lens. https://www.agisoft.es/products/agisoft-lens-2/.

[41] Artec 3D Artec Studio 11 (Software). Version 2016. http://www.artec3.com/.

[42] Agisoft PhotoScan 2016 (Software). Version 2016. http://www.agisoft.com/.

[43] Sagawa R., Ota Y., Yagi Y., Furukawa R., Asada N., Kawasaki H. Dense 3D reconstruction method using a single pattern for fast moving object. Proceedings of the 2009 IEEE 12th International Conference on computer Vision.

[44] (2016). Cloud Compare (Version 2.7.0) (GPL Software). http://www.cloudcompare.org/.

