# Supplementary material for: Low-Cost Prototype to Automate the 3D Digitization of Pieces: An Application Example and Comparison
Source: Sensors (Basel). 2021 Apr 7;21(8):2580. doi: 10.3390/s21082580 (PMC8067622; doi:10.3390/s21082580)
Supplement: Supplementary file 1 [file sensors-21-02580-s001.pdf]

sensors MDPI

Supplementary Materials

General specifications	Spider
3D resolution, up to	0.1 mm
3D point accuracy, up to	0.05 mm
3D accuracy over distance, up to	0.03% over 100 cm
Colours	24 bpp
Texture resolution	1.3 mp
Light source	Blue LED
Working distance	0.17–0.35 m
Linear field of view, HxW @ closest range	90 × 70 mm
Linear field of view, HxW @ furthest range	180 × 140 mm
Angular field of view, HxW	30 × 21°
Video frame rate, up to	7.5 fps
Exposure time	0.0005 s
Data acquisition speed, up to	1 mln points/sec.
Multi-core processing	Yes
**Output formats**	
3D Formats	OBJ, PLY, WRL, STL, AOP, ASCII, Disney PTEX, E57, XYZRGB
Formats for measurements	CSV, DXF, XML
**Common specifications**	
Weight	0.85 kg
Dimensions, HxDxW	190 × 140 × 130 mm
Power consumption	12V, 24W
Interface	1 × USB 2.0, USB 3.0 compatible
Processing capacity	40 mln triangles/ 1 GB RAM
**Requirements**	
Processor	Intel I5 or I7
Memory	18 GB
Video cards	NVIDIA, AMD or Intel
OS	Windows 7, 8, 10 – x64
Free disk space	300 MB for installation

Table S1. 3D scanner Spider Artec
(Source: www.artec3d.com)

**Specifications**	**Nikon D5100**
Type	Single-lens reflex digital camera
Lens mount	Nikon F mount (with AF contacts)
Effective angle of view	Approx. 1.5 x lens focal length (Nikon DX format)
Effective pixels	16.2 million
Image sensor	Sensor CMOS de 23.6 x 15.6mm
Total pixels	16.9 million
Dust-reduction system	Image Sensor Cleaning, Airflow Control System, Image Dust Off reference data (optional Capture NX 2 software required)
Image size (pixels)	4.928 x 3.264 (L); 3.696 x 2.448 (M); 2.464 x 1.632 (S)
File format	NEF (RAW): 14 bit, compressed<br>JPEG: JPEG-Baseline compliant with fine (approx. 1:4), normal (approx. 1:8) or basic (approx. 1:16) compression<br>NEF (RAW) + JPEG: Single photograph recorded in both NEF (RAW) and JPEG formats
Picture Control System	Standard, Neutral, Vivid, Monochrome, Portrait, Landscape; selected Picture Control can be modified; storage for custom Picture Controls
Media	SD (Secure Digital), SDHC and SDXC memory cards
File system	DCF (Design Rule for Camera File System) 2.0, DPOF (Digital Print Order Format), Exif 2.3 (Exchangeable Image File Format for Digital Still Cameras), PictBridge
Viewfinder	Eye-level pentamirror single-lens reflex viewfinder
Magnification	approx. 0.78
Shutter type	Electronically-controlled vertical-travel focal-plane shutter
Shutter speed	1/4.000 to 30 s
Self-timer	2 s, 5 s, 10 s, 20 s; de 1 to 9 exposures
Metering method	Matrix, Centre-weighted or Spot
ISO sensitivity	ISO 100 to 6400 in steps of 1/3 EV
File format video	MOV

Table S2. Nikon camera characteristics
(Source: www.nikon.es)
